# Fabrication and in vivo 2-photon microscopy validation of transparent PEDOT:PSS microelectrode arrays

**DOI:** 10.1038/s41378-022-00434-7

**Published:** 2022-08-29

**Authors:** Gerwin Dijk, Attila Kaszas, Jolien Pas, Rodney Philip O’Connor

**Affiliations:** 1 Mines Saint-Etienne, Centre CMP, Department of Bioelectronics, Gardanne, 13541 France; 2 Panaxium SAS, Aix-en-Provence, 13100 France

**Keywords:** Engineering, Materials science

## Abstract

Transparent microelectrode arrays enable simultaneous electrical recording and optical imaging of neuronal networks in the brain. Electrodes made of the conducting polymer poly(3,4-ethylenedioxythiophene) doped with polystyrene sulfonate (PEDOT:PSS) are transparent; however, device fabrication necessitates specific processes to avoid deterioration of the organic material. Here, we present an innovative fabrication scheme for a neural probe that consists of transparent PEDOT:PSS electrodes and demonstrate its compatibility with 2-photon microscopy. The electrodes show suitable impedance to record local field potentials from the cortex of mice and sufficient transparency to visualize GCaMP6f-expressing neurons underneath the PEDOT:PSS features. The results validate the performance of the neural probe, which paves the way to study the complex dynamics of in vivo neuronal activity with both a high spatial and temporal resolution to better understand the brain.

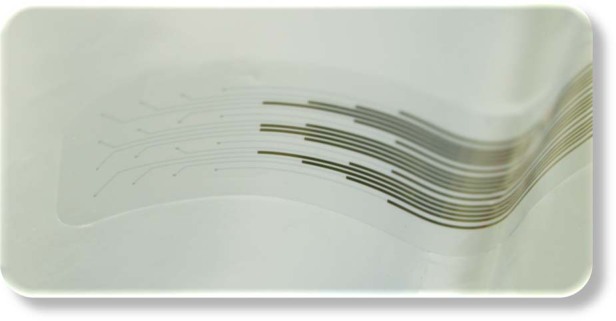

## Introduction

The complexity of neuronal networks has pushed the development of advanced tools and techniques to study the nervous system^1,2^. Microelectrode arrays (MEAs) have been widely used to record or evoke electrical activity of neurons; however, electrodes as small as 20 µm in diameter still lack spatial information since they are on the same order of magnitude as the size of cells^3^. Combining electrical interfaces with advanced imaging techniques could provide additional, high-resolution, visual data regarding cellular and network structures and specific biomarkers^4^.

Integration of high-density MEAs, such as electrocorticography (ECoG) devices, with imaging setups requires optical transparency of the electrodes and interconnects. Materials such as graphene^5–7^, carbon nanotubes^8^ and indium tin oxide (ITO)^9,10^ have shown adequate transparency, but their electrochemical performance is compromised by the relatively high electrode impedance caused by the double layer capacitance at the electrode-electrolyte interface. The conducting polymer poly(3,4-ethylenedioxythiophene) doped with polystyrene sulfonate (PEDOT:PSS) has shown adequate transparency and facilitates electronic and ionic conduction, which greatly enhances the electrochemical interaction with electrolytes and reduces the electrode impedance^11,12^. Moreover, it can be processed from solution^13^, provides a good interface for assessing biological functions^14,15^, and has shown stable performance for four months in cell culture conditions^16^.

Despite the needed electrochemical and optical properties of PEDOT:PSS, the fabrication of devices that include the patterning of such organic materials requires specific processes to avoid material deterioration. Printing might be a viable technique but requires ink formulations with particular rheological properties and offers limited resolution^17^. Alternatively, thin-film patterning methods based on photolithography and well-established PEDOT:PSS deposition protocols are available^13,18–20^. Most of these approaches, however, apply PEDOT:PSS as a coating for the underlying metallic electrodes and are not suitable for devices in which both the interconnects and electrodes consist of PEDOT:PSS only. For instance, electrochemical deposition cannot be utilized due to the absence of a conductor that can function as the working electrode^18^. Photolithographic patterning of PEDOT:PSS without the need for an underlying metallic layer can be achieved with a sacrificial Parylene C peel-off method in combination with spin coating a dispersion^19,20^; however, the opening of the encapsulation at the electrode sites in the subsequent step would require etching, which could deteriorate the PEDOT:PSS. These limitations necessitate alternative manufacturing methods based on compatible processes and chemicals that do not damage the organic materials.

Here, we present a thin-film microfabrication process for a transparent and conformal ECoG device that contains PEDOT:PSS electrodes and interconnects that can be used in combination with imaging setups. The process flow is based on an ‘upside-down’ approach, where the electrodes are facing the carrier. The electrical functionality of the electrodes was determined with electrochemical impedance spectroscopy (EIS), and bright field images of cultured neurons on the electrodes were used to demonstrate the optical transparency. Finally, the device was implanted on the cerebral cortex of a mouse to record local field potentials (LFPs) and demonstrate 2-photon microscopy compatibility.

## Materials and methods

### Fabrication process

**Fig. 1:**
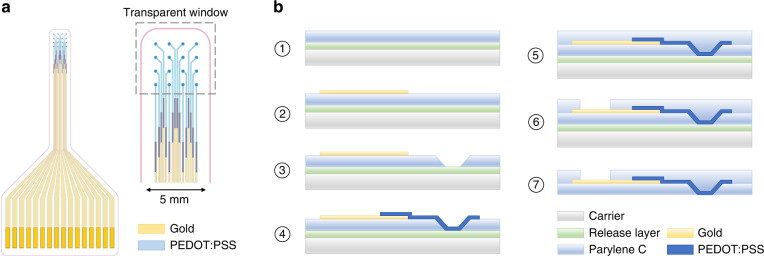
Design and fabrication of the transparent neural probe. **a** Device layout with 16 PEDOT:PSS electrodes and 2.5 mm long PEDOT:PSS interconnects. Outside the transparent window gold interconnects were used to minimize resistive losses. **b** Schematic representation of the fabrication process which included the use of a release layer, opening of the electrode sites, gold and PEDOT:PSS patterning, and encapsulation with Parylene C.

Figure 1 shows the design of the neural probe and the fabrication process. The Parylene C encapsulated probe contained 16 PEDOT:PSS electrodes that were arranged in a 4 × 4 matrix with a spacing of 600 µm between the electrodes. The interconnects had a total length of 30 mm, of which 27.5 mm consisted of gold and 2.5 mm consisted of PEDOT:PSS. The 12 mm overlap between the gold and PEDOT:PSS interconnects ensured proper electrical connection. All PEDOT:PSS interconnects were 2.5 mm in length to obtain the same resistive loss for each electrode. This design resulted in a neural probe with a transparent window at the tip that contained solely Parylene C and PEDOT:PSS.

The fabrication process was designed such that the active electrode sites were facing the carrier, which necessitated the use of a PEDOT:PSS-compatible release layer. Here, we used a fluorinated photoresist that is commonly used for orthogonal processing of organic conducting materials^21^. The fabrication of the neural probes contained the following 7 steps:

1. After cleaning of the glass carrier, a 3 µm layer of fluorinated photoresist was spin coated followed by the deposition of a 2 µm layer of Parylene C.
2. Metal leads were patterned through a lift-off process involving a 4 µm AZ nLOF 2070 photoresist layer and thermal deposition of 10 nm of titanium and 120 nm of gold.
3. The active electrode sites were opened by reactive ion etching with AZ 10XT photoresist as an etch mask. The etching time was accurately controlled to ensure complete removal of the Parylene C while marginally etching the underlying fluorinated photoresist. Since AZ 10XT strippers are not compatible with the fluorinated photoresist, any remaining photoresist could not be stripped. Therefore, the thickness of AZ 10XT was precisely controlled such that no resist remained after etching. Parylene C and AZ 10XT showed similar etch rates, therefore requiring a 2 µm layer of photoresist for 2 µm of Parylene C. We used edge bead remover (EBR) solvent to dilute AZ 10XT and calibrated the thickness with a spin curve. AZ 10XT was exposed with a 40 µm gap between the substrate and mask to create an overcut profile, that was transferred into the Parylene C by the etch process. The features with an overcut profile established a smooth Parylene C topology, which was needed to deposit a continuous PEDOT:PSS film, i.e., where the sidewalls of the Parylene C structure are coated as well.
4. PEDOT:PSS interconnects and electrodes were patterned through dry etching. First, 4 layers of a PEDOT:PSS dispersion containing Clevios PH1000, glycerol, dodecyl benzene sulfonic acid, and (3-glycidyloxypropyl)trimethoxysilane were spin coated and annealed. The stacking of multiple PEDOT:PSS layers is a validated process to obtain thicker films^22^. The obtained thickness of ~500 nm has been shown to be a good compromise between a low electrode impedance and a sufficient transparency with a transmittance of ~70 % for 940 nm, which is the relevant wavelength for 2-photon imaging^12^. Next, 10% poly(vinyl alcohol) was dissolved in deionized water, spin coated and dried followed by spin coating, exposure and development of a 4.5 µm layer of fluorinated photoresist. After reactive ion etching, the remaining photoresist was stripped followed by immersion in deionized water. Note that the large sidewall angle of the Parylene C etch aids the PEDOT:PSS coating of the sidewalls.
5. Encapsulation was finished by deposition of a 2 µm layer of Parylene C.
6. Connection pads and the outline of the probe were opened by reactive ion etching with a patterned AZ 10XT photoresist etch mask.
7. The device was released by immersing the carrier in the fluorinated photoresist stripper.

Crucial aspects in this fabrication process were (a) the use of a release layer that is compatible with organic materials and (b) a smooth etch topology of the parylene C encapsulation layer. Additionally, this fabrication scheme resulted in a neural interface with the connection pads and electrode sites facing the opposite side.

### Electrochemical characterization

EIS was performed with a PalmSens4 potentiostat in phosphate-buffered saline (PBS) employing a 3-electrode setup. The PEDOT:PSS electrode, a platinum mesh and an Ag/AgCl electrode functioned as the working, counter, and reference electrode, respectively. A 25 mV sinusoidal voltage was applied between 1 and 100000 Hz with 4 measurements per decade. EIS was performed on the electrodes of the neural probe, as well as on test devices with PEDOT:PSS interconnect lengths of 0.5, 2, 5, and 10 mm (electrode diameter 100 µm) and electrode diameters of 50, 100, 250, 500, and 1000 µm (PEDOT:PSS interconnect length 2.5 mm).

### Scanning electron microscope

Scanning electron microscopy (SEM) images were acquired after fabrication steps 4 and 7 with a Carl Zeiss Ultra 55 after deposition of 5 nm gold–palladium.

### In vitro cortical cell culture

To demonstrate transparency for biological samples, neurospheres of cortical cells were cultured on the electrode array employing a previously reported protocol^23^. In short, devices were sterilized and coated with poly-D-lysine and laminin. Cells were dissociated from E15 rat cortical tissue (NeuroSYS) with papain solution (Hibernate EB, Brainbits, LLC) and plated at a cell density of 900 cells mm^−2^. Cells were cultured for 4 days in Neurocult neuronal plating media with 2% NeuroCult SM1 Supplement (Stemcell Technologies), 500 µM GlutaMax (Gibco), and 25 µM glutamic acid (Sigma Aldrich), after which half of the media was replaced every 2–3 days with BrainPhys neuronal medium containing 2% Neurocult SM1 (Stemcell Technologies). Bright field images were taken after 8 days in vitro.

### In vivo implantation and 2-photon microscopy

The neural probes were implanted in GCaMP6f-expressing mice to acquire LFP recordings and demonstrate 2-photon transparency. Surgical methods were conducted as described previously^4,24^. Briefly, animals were gas anesthetized (2.5% sevoflurane), a 5 mm diameter cranial window was opened above the right hemisphere cortices, and the transparent probe was placed onto the dura. The probe was covered with a glass coverslip (WPI) that was fixed with light-curing dental resin. LFP recordings were acquired with an Intan RHS controller and 2-photon imaging was performed with a FemtoSmart Dual microscope (Femtonics Ltd, Budapest, Hungary). The anesthetized (0.9% isoflurane) head-fixed animals were imaged using a 16x objective (Nikon LWD 16x/0.8 NA) and 940 nm pulsed laser light (MaiTai HP, SpectraPhysics).

## Results

### Transparent conformal microelectrode array

**Fig. 2:**
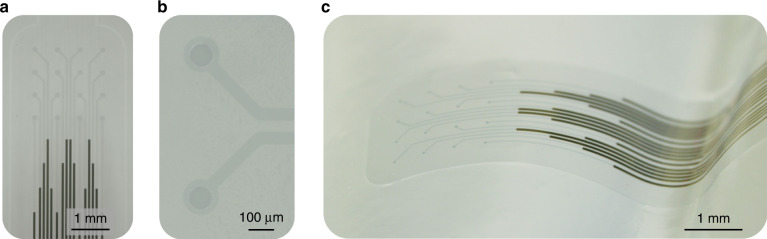
Transparent and conformable PEDOT:PSS neural probe. **a** Optical image of the probe containing 16 PEDOT:PSS electrodes. The gold interconnects are visible at the bottom of the image. **b** Magnified image of two electrodes. **c** Image of the probe placed on a curved agarose brain model.

Figure 2a, b show the tip of a fabricated neural probe and two of the electrodes. The tip of the probe was 3 × 3 mm^2^, which are suitable dimensions for implantation on the cortex of a mouse. This part of the probe contained the PEDOT:PSS interconnects and electrodes that provide a transparent window.

The excellent conformability of the neural probe was verified by placing the neural probe on a curved agarose brain model (Fig. 2c). Conformability is a crucial requirement for biocompatible and stable implants because it reduces the foreign body response. In addition to the Young’s modulus of the material, the thickness of the device plays a crucial role in reliably conforming the device to the tissue curvature^25^. The total thickness of our probe was ~4 µm, which resulted in a highly flexible and very conformable device.

**Fig. 3:**
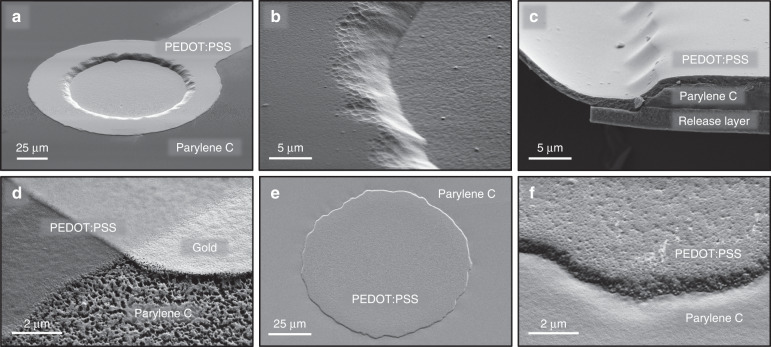
Tilted SEM of PEDOT:PSS electrodes and interfaces. **a**–**d** Micrographs after completion of PEDOT:PSS patterning (fabrication step 4). To obtain the cross section in (**c**), the device was cut and released from the carrier. **e** and **f** Micrographs after the fabrication was completed.

SEM images were acquired after completion of fabrication steps 4 and 7 to verify and optimize the processes. Figure 3a–d show SEM images of the device after completion of fabrication step 4. At this point, the electrode openings were etched, and the gold and PEDOT:PSS were patterned. The micrographs in Fig. 3a–c confirm a continuous PEDOT:PSS layer on both the underlying Parylene C and fluorinated resist. Moreover, the overcut etch profile of the Parylene C was properly coated, ensuring electric connection from the PEDOT:PSS electrode site to the PEDOT:PSS interconnect. Figure 3d shows a micrograph of the gold and PEDOT:PSS interconnects. Electrical contact is established by the continuous PEDOT:PSS interconnect that overlaps the gold. Figure 3e, f show micrographs of the electrode after the release of the device from the carrier. The surface of the PEDOT:PSS electrode was situated in the same plane as the Parylene C and showed a tight PEDOT:PSS-Parylene C interface. A slight PEDOT:PSS roughness was observed, which was imprinted by the underlying fluorinated resist that was roughened by the etching process.

### Electrochemical characterization

**Fig. 4:**
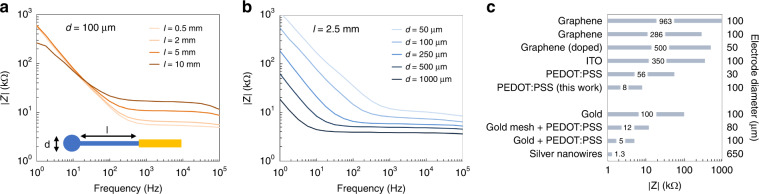
EIS for electrodes with various design parameters. **a** Impedance for various PEDOT:PSS interconnect lengths (100 µm electrode diameter). **b** Impedance for various electrode diameters (2.5 mm PEDOT:PSS interconnect length). **c** Reported electrode impedances at 1 kHz for various transparent electrodes made of materials such as graphene^6,7^, doped graphene^5^, ITO^10^, PEDOT:PSS^12^, gold^27^, gold coated with PEDOT:PSS^27,28^, and silver^29^. Note the difference in diameter for some of the electrodes. The condition labeled “PEDOT:PSS (this work)” represents the impedance value of a 100 µm electrode with a 2.5 mm interconnect.

The electrochemical performance of the electrodes was characterized by EIS. Figure 4a, b show the magnitude of the electrode impedance (|Z|) for various electrode diameters and PEDOT:PSS interconnect lengths. All EIS measurements displayed typical PEDOT:PSS impedance behavior with a resistance-dominated region at high frequencies separated by a cutoff frequency from a capacitance-dominated region at lower frequencies. Long PEDOT:PSS interconnects showed higher impedances in the high-frequency domain, including 1 kHz, which is the frequency of interest for recording action potentials. The increased impedance is caused by the 1.4 kΩ/mm resistance of the PEDOT:PSS interconnect, a value that was determined by measuring the resistance of PEDOT:PSS interconnects with various lengths. For recording and imaging of biological samples, neural probes were used with a PEDOT:PSS interconnect of 2.5 mm, resulting in a resistance of 3.5 kΩ. Electrodes with a smaller diameter showed higher impedance over the full frequency range. This corresponds with previous findings where the electrode capacitance scales linearly with the electrode surface area and the spreading resistance is inversely proportional to the electrode diameter^26^. Overall, the EIS of the electrodes displayed the general characteristics of PEDOT:PSS electrodes, which confirms electrode functionality and validates the fabrication process.

For comparison, Fig. 4c shows the 1 kHz electrode impedance of various electrodes as previously reported in the literature. The PEDOT:PSS electrodes in this work exhibited an impedance that is (1) almost two orders of magnitude lower than other transparent materials such as graphene and ITO, (2) an order of magnitude lower than gold, and (3) similar to gold electrodes coated with PEDOT:PSS. Note that the difference between electrodes made of PEDOT:PSS (8 kΩ) and those made of gold coated with PEDOT:PSS (5 kΩ) corresponds to the resistance of the interconnect as mentioned above (3.5 kΩ).

### Imaging and recording of biological samples

**Fig. 5:**
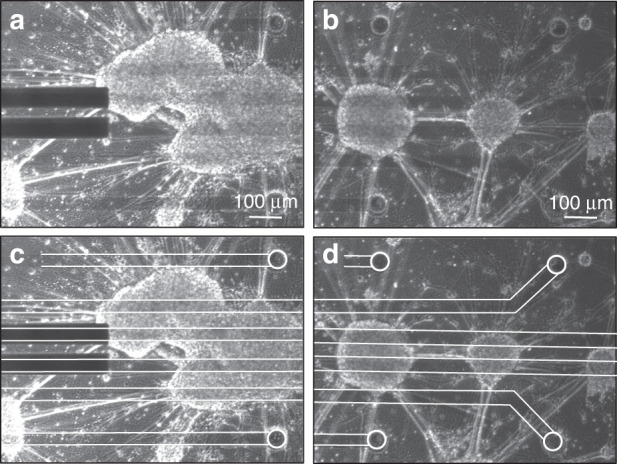
Bright field images of clustered neuronal cells cultured on top of the neural probe. **a** and **b** Neuronal networks cultured on top of the device. **c** and **d** Same images as (**a**) and (**b**), respectively, with the white lines and circles indicating the location of the 50 µm PEDOT:PSS interconnects and electrodes.

The visibility of biological systems through the electrodes was first verified by culturing cortical neurospheres containing neuronal cells on top of the device (Fig. 5) Complex neuronal networks were clearly visible, and nearly no visual obstruction from the PEDOT:PSS was observed while imaging through the conducting layer. In contrast, the gold interconnects are opaque, hindering the optical visualization of cellular features.

**Fig. 6:**
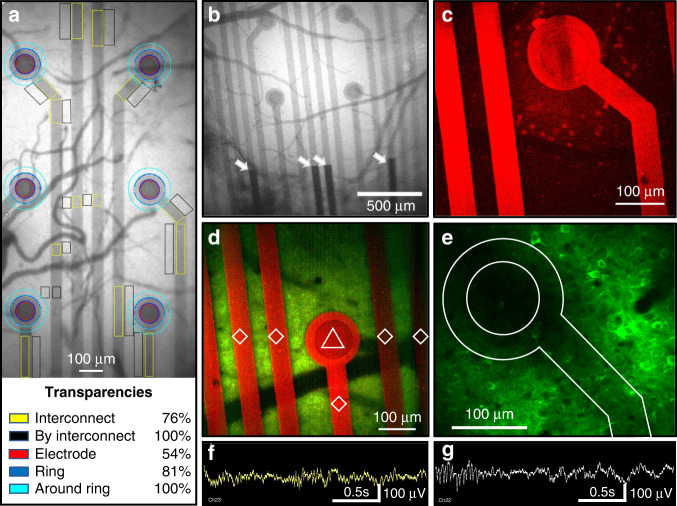
Imaging and LFP recordings of the neural probe on the cortex of a mouse. **a** Infrared image of the probe on the cortex (6 out of 16 electrodes are shown). The colored regions of interest indicate the areas used to calculate the PEDOT:PSS transparencies values, which are based on the gray levels compared to adjacent areas that are not covered by PEDOT:PSS. **b** Infrared image of the probe on the cortex (4 out of 16 electrodes are shown); the white arrows indicate the gold interconnects. **c** and **d** 2-photon images with the focus plane on the probe. **e** 2-photon image with the focus plane 270 µm beneath the probe. **f** and **g** LFP recordings from the electrodes in (**d**) and (**e**), respectively.

For in vivo implantation and testing, the placement of the probes was first validated using an infrared camera (Fig. 6a, b). The gold interconnects prevented the transmission of all light, whereas blood vessels under the PEDOT:PSS could clearly be located and visualized. The infrared images also showed different levels of transparency between the circular electrodes and the interconnect lines. Therefore, we quantified the transparency for various regions by comparing the mean gray values from specific parts of the probe that contained PEDOT:PSS with adjacent regions without PEDOT:PSS. The transparency of the electrodes, the rings around the electrodes, and the interconnect lines was 54% (*n* = 6 electrodes), 81% (*n* = 6 rings), and 76% (*n* = 14 regions), respectively. Figure 6c, d show 2-photon images with the neural probe in the focal plane. The PEDOT:PSS layer appeared highly fluorescent, as reported in recent studies^30^. On the other hand, when the focal plane was adjusted to 270 µm beneath the probe, green GCaMP6f-expressing neurons could be observed (Fig. 6e). Importantly, the 50 µm wide PEDOT:PSS interconnects cast only a minor shadow at this depth, allowing clear visualization of neuronal somata and neuronal processes.

The transparent PEDOT:PSS probes have also been tested in LFP recordings from the visual cortical surface of mice in vivo. As shown in Fig. 6f, g, the amplitude of signals reached 100 µV and with relatively low levels of noise. These experiments demonstrate adequate LFP recordings in combination with 2-photon imaging.

## Discussion

Low-impedance, transparent PEDOT:PSS electrode arrays were fabricated using a novel fabrication procedure with the electrode sites facing the carrier. The procedure contains two critical aspects to overcome the challenge of patterning and encapsulating PEDOT:PSS electrodes without deteriorating the material. First, we used a fluorinated photoresist as a release layer, which enabled such meticulous fabrication due to its unique properties. The release layer needs to be (1) compatible with PEDOT:PSS to avoid deterioration, (2) easy to dissolve in a stripper for device release from the carrier, and (3) reasonably thick and etch resistant to avoid contact of the PEDOT:PSS layer with the carrier. In addition to a fluorinated photoresist, there might be other materials available that fulfill these requirements and could function as the release layer. Second, to ensure a continuous PEDOT:PSS coating over the Parylene C topology, angled sidewalls were designed and optimized using gap exposure of the photoresist. Although isotropic etching might also result in such profiles, we achieved larger sidewall angles with gap exposures. A PEDOT:PSS thickness of ~500 nm was chosen, as it was previously determined to have ~75% transparency in the visual spectrum. Although thinner films would have increased transparency, the increased electrode impedance would compromise the recording quality^12^. The proposed method results in an optically transparent electrode array with an electrode impedance that is almost two orders of magnitude lower than that of ITO or graphene electrodes.

Design parameters such as electrode size, interconnect width, and interconnect length have a significant influence on the electrode impedance. The probe presented here had a minimum feature size of 50 μm; however, high-density electrode arrays might require narrower interconnects and smaller electrodes. This would increase the electrode impedance since the interconnect resistance scales inversely with the cross section according to *R* = *ρl*/*A*. Moreover, reducing the electrode diameter decreases the capacitance and increases the spreading resistance. Ultimately, the smallest feature size is limited by the employed fabrication process and is on the order of 10 µm. This limit is comparable to most commonly used fabrication schemes, including when PEDOT:PSS is used to coat gold or platinum electrodes, because these rely on the same equipment and similar processes such as lithography, lift-off, and etching^20^. The PEDOT:PSS interconnect length is an important design parameter since long interconnects increase the electrode impedance. To avoid resistive losses, the PEDOT:PSS interconnects should be designed as short as possible with metallic leads underneath once outside the imaging area of interest. To ensure comparable impedances for all electrodes, the PEDOT:PSS interconnects should have equal lengths.

The multimodal approach to study biological systems both electrically and optically holds great potential. In this work, we validated the in vivo electrical recording and 2-photon imaging separately. However, previously reported recordings and imaging acquired with PEDOT:PSS electrode arrays suggest that combining 2-photon imaging and LFP recordings is feasible^4,12^. High levels of autofluorescence caused by photoluminescence were observed when the focus plane of the microscope corresponded to the PEDOT:PSS layer on the device. We showed that this can be avoided by adjusting the focus plane beneath the electrodes. Alternatively, imaging in the same plane as the probe is possible when the scanning laser remains at >50 µm from the electrodes and interconnects^31^.

Overall, we demonstrated a microfabrication process to manufacture microelectrode arrays to both optically and electrically assess neuronal activity from the surface of the brain. In addition to surface electrodes, we have previously explored other neural interfacing methods, such as penetrating probes that contain electrodes, for neuronal recordings, stimulation, and drug delivery^32–34^. The transparent probe microfabrication presented in this work could be adapted to such penetrating devices, potentially opening the way for electrophysiological recordings and stimulation combined with whole field-of-view imaging at depth. Other applications might be found in systems neuroscience and connectomics, or anywhere investigators wish to simultaneously acquire multielectrode extracellular electrophysiology and multiphoton imaging data.

## References

[1] Minev IR (2015). Electronic dura mater for long-term multimodal neural interfaces. Science.

[2] Klapoetke NC (2014). Independent optical excitation of distinct neural populations. Nat. Methods.

[3] Khodagholy D (2011). Highly conformable conducting polymer electrodes for in vivo recordings. Adv. Mater..

[4] Donahue MJ (2018). Multimodal characterization of neural networks using highly transparent electrode arrays. eNeuro.

[5] Kuzum D (2014). Transparent and flexible low noise graphene electrodes for simultaneous electrophysiology and neuroimaging. Nat. Commun..

[6] Park D-W (2018). Electrical neural stimulation and simultaneous in vivo monitoring with transparent graphene electrode arrays implanted in GCaMP6f mice. ACS Nano.

[7] Thunemann M (2018). Deep 2-photon imaging and artifact-free optogenetics through transparent graphene microelectrode arrays. Nat. Commun..

[8] Zhang J (2018). Stretchable transparent electrode arrays for simultaneous electrical and optical interrogation of neural circuits in vivo. Nano Lett..

[9] Zátonyi A (2018). Functional brain mapping using optical imaging of intrinsic signals and simultaneous high-resolution cortical electrophysiology with a flexible, transparent microelectrode array. Sens. Actuators, B Chem..

[10] Zátonyi A (2020). Transparent, low-autofluorescence microECoG device for simultaneous Ca^2+^ imaging and cortical electrophysiology in vivo. J. Neural Eng..

[11] Kshirsagar P (2019). Transparent graphene/PEDOT:PSS microelectrodes for electro- and optophysiology. Adv. Mater. Technol..

[12] Middya S (2021). Microelectrode arrays for simultaneous electrophysiology and advanced optical microscopy. Adv. Sci..

[13] Donahue MJ (2020). Tailoring PEDOT properties for applications in bioelectronics. Mater. Sci. Eng. R: Rep..

[14] Zeglio E, Rutz AL, Winkler TE, Malliaras GG, Herland A (2019). Conjugated polymers for assessing and controlling biological functions. Adv. Mater..

[15] Dijk G, Poulkouras R, O’Connor RP (2022). Electroporation microchip with integrated conducting polymer electrode array for highly sensitive impedance measurement. IEEE Trans. Biomed. Eng..

[16] Dijk G, Rutz AL, Malliaras GG (2020). Stability of PEDOT:PSS‐coated gold electrodes in cell culture conditions. Adv. Mater. Technol..

[17] Yuk H (2020). 3D printing of conducting polymers. Nat. Commun..

[18] Cui X, Martin DC (2003). Electrochemical deposition and characterization of poly(3,4-ethylenedioxythiophene) on neural microelectrode arrays. Sens. Actuators B Chem..

[19] DeFranco JA, Schmidt BS, Lipson M, Malliaras GG (2006). Photolithographic patterning of organic electronic materials. Org. Electron..

[20] Sessolo M (2013). Easy-to-fabricate conducting polymer microelectrode arrays. Adv. Mater..

[21] Taylor PG (2009). Orthogonal patterning of PEDOT:PSS for organic electronics using hydrofluoroether solvents. Adv. Mater..

[22] Dijk G, Ruigrok HJ, O’Connor RP (2020). Influence of PEDOT:PSS coating thickness on the performance of stimulation electrodes. Adv. Mater. Interfaces.

[23] Pas J (2018). Neurospheres on patterned PEDOT:PSS microelectrode arrays enhance electrophysiology recordings. Adv. Biosyst..

[24] Kaszas A (2021). Two-photon GCaMP6f imaging of infrared neural stimulation evoked calcium signals in mouse cortical neurons in vivo. Sci. Rep..

[25] Vomero M (2020). Conformable polyimide-based μECoGs: Bringing the electrodes closer to the signal source. Biomaterials.

[26] Koutsouras DA (2017). Impedance spectroscopy of spin‐cast and electrochemically deposited PEDOT:PSS films on microfabricated electrodes with various areas. ChemElectroChem.

[27] Ganji, M. et al. Scaling effects on the electrochemical performance of poly(3,4‐ethylenedioxythiophene (PEDOT), Au, and Pt for electrocorticography recording. Adv. Funct. Mater. 27, 1703018 (2017).

[28] Qiang, Y. et al. Transparent arrays of bilayer-nanomesh microelectrodes for simultaneous electrophysiology and two-photon imaging in the brain. *Sci. Adv.* **4**. http://advances.sciencemag.org/ (2018). 10.1126/sciadv.aat0626 PMC6124910 30191176

[29] Tian J (2021). Stretchable and transparent metal nanowire microelectrodes for simultaneous electrophysiology and optogenetics applications. Photonics.

[30] Marek T (2021). Optimization aspects of electrodeposition of photoluminescent conductive polymer layer onto neural microelectrode arrays. Mater. Chem. Phys..

[31] Renz AF (2020). Opto‐E‐Dura: a soft, stretchable ECoG array for multimodal, multiscale neuroscience. Adv. Healthc. Mater..

[32] Pas J (2018). A bilayered PVA/PLGA-bioresorbable shuttle to improve the implantation of flexible neural probes. J. Neural Eng..

[33] Williamson A (2015). Localized neuron stimulation with organic electrochemical transistors on delaminating depth probes. Adv. Mater..

[34] Proctor, C. M. et al. Electrophoretic drug delivery for seizure control. *Sci. Adv.* **4**, eaau1291 (2018). 10.1126/sciadv.aau1291 PMC6114990 30167463

